# Patient Preferences for Patient Portal–Based Telepsychiatry in a Safety Net Hospital Setting During COVID-19: Cross-sectional Study

**DOI:** 10.2196/33697

**Published:** 2022-01-26

**Authors:** Han Yue, Victoria Mail, Maura DiSalvo, Christina Borba, Joanna Piechniczek-Buczek, Amy M Yule

**Affiliations:** 1 Department of Psychiatry Boston Medical Center Boston, MA United States; 2 Department of Psychiatry Massachusetts General Hospital Boston, MA United States; 3 Department of Psychiatry Boston University School of Medicine Boston, MA United States

**Keywords:** patient portal, telemedicine, telepsychiatry, COVID-19, portal, mental health, psychiatry, engagement, behavior, video, hospital, urban, outreach

## Abstract

**Background:**

Patient portals are a safe and secure way for patients to connect with providers for video-based telepsychiatry and help to overcome the financial and logistical barriers associated with face-to-face mental health care. Due to the COVID-19 pandemic, telepsychiatry has become increasingly important to obtaining mental health care. However, financial and technological barriers, termed the “digital divide,” prevent some patients from accessing the technology needed to use telepsychiatry services.

**Objective:**

As an extension to a clinic’s outreach project during COVID-19 to improve patient engagement with video-based visits through the hospital’s patient portal among adult behavioral health patients at an urban safety net hospital, we aim to assess patient preference for patient portal–based video visits or telephone-only visits and to identify the demographic variables associated with their preference.

**Methods:**

Patients in an outpatient psychiatry clinic were contacted by phone, and preference for telepsychiatry by phone or video through a patient portal, as well as device preference for video-based visits, were documented. Patient demographic characteristics were collected from the electronic medical record.

**Results:**

A total of 128 patients were reached by phone. A total of 79 (61.7%) patients chose video-based visits, and 69.6% (n=55) of these patients preferred to access the patient portal through a smartphone. Older patients were significantly less likely to agree to video-based visits.

**Conclusions:**

Among behavioral health patients at a safety net hospital, there was relatively low engagement with video-based visits through the hospital’s patient portal, particularly among older adults.

## Introduction

There has been a rapid shift in the delivery of outpatient medical and mental health care from in-person to virtual through teleconferencing services/platforms since the World Health Organization declared the COVID-19 outbreak a global pandemic on March 11, 2020 [1]. Virtual mental health care and telepsychiatry encompasses care provided through various electronic means, often involving live video and audio, but also including telephone-only visits [2]. Prior to COVID-19, telepsychiatry use by phone and video was highly region-dependent and was used more in rural and Health Profession Shortage Areas in which geographic isolation and provider shortages created difficulties in accessing face-to-face mental health care [2,3]. For example, between 2014 and 2016, only 0.1% of psychiatrists in Massachusetts performed telepsychiatry work, compared to 24.2% of psychiatrists doing so in North Dakota [2]. To support safe and accessible health care services, the Centers for Medicare and Medicaid Services expanded coverage for telemedicine and telepsychiatry services at the beginning of the COVID-19 pandemic by declaring that it would reimburse video and audio-only evaluation and management services at rates equivalent to in-person visits. As a result of these changes, by the end of March, most hospitals in the United States had begun offering virtual appointments for their patients [2]. A subsequent June 2020 national survey found that 85% of psychiatrists were seeing most of their patients through telepsychiatry [4].

In addition to helping limit viral spread, telepsychiatry carries other advantages for both the patient and provider. For patients, telepsychiatry enables access to mental health services from their own homes, which for some, decreases anxiety and stress associated with leaving a safe and private environment, and allays financial burdens associated with transportation and taking time off from work [2,5,6]. From a provider standpoint, clinical care can be enhanced through greater understanding of the patient’s family and living situation [2]. Furthermore, studies have suggested that telepsychiatry assessments are as effective as in-person visits across various psychiatric diagnoses and patient populations [2,5]. Telepsychiatry can occur over the phone or by video, although video-based appointments have been described by clinicians as superior to phone-only visits for providing reassurance, building rapport, and gathering greater clinical information [7].

Although telepsychiatry provides many advantages in the current environment, successful use of these services requires a consistent level of technological access that may be prohibitive for some patients. A 2019 Pew Research Center report found that, although most Americans are able to access the internet through laptops, desktops, and tablets, lower educational attainment and lower annual income were associated with decreased access to these types of devices and to broadband internet access [8]. Currently, at least 21 million Americans lack consistent access to broadband internet, which results in a reliance on mobile devices to access internet services [9]. Although most Americans have smartphones, 17% of Americans are “smartphone-dependent,” relying almost exclusively on smartphones for online access. The number of “smartphone-dependent” individuals has also approximately doubled since 2013 [8,10]. Increasingly, mobile devices are the primary method of internet access for vulnerable populations [8,10,11]. The impact of the “digital divide,” defined as the inequality between those who have access to devices and the internet and those who do not, on health, education, and employment has been previously recognized [8,11,12]. However, the COVID-19 pandemic has made these inequities more evident due to the greater demand and need for fast, reliable access to the internet [13]. More than ever before, the internet has become essential for allowing patients to interface with the mental health system. In many health care systems, this rapid expansion of telepsychiatry services during the COVID-19 pandemic did not allow for adequate evaluation of barriers to telepsychiatry use or preparation of a plan to manage these barriers [11]. An inability to adequately address these issues may result in further disparities and a widening of the “digital divide” [11].

One means by which telepsychiatry visits can occur is through online patient portals. Patient portals are secure locations on the internet from which patients can access personal health information, schedule appointments, communicate with their providers, and connect with providers for video-based appointments [14-16]. In behavioral health settings, patient portals have been shown to increase a sense of patient autonomy, improve patient activation, and decrease administrative inefficiencies [17,18]. A recent study in a large health system across multiple specialties has also demonstrated patient portal activation is associated with increased patient ability to complete telemedicine visits [19]. Furthermore, video-based appointments performed directly through electronic medical record (EMR)–based patient portals such as MyChart provide an additional layer of security compared to separate virtual platforms such as Doximity or Skype [15] and have the advantage of closer integration with the patient’s medical record, which may help with documentation and billing [20]. Unfortunately, direct comparisons between different virtual platforms are limited. Notably, individuals who access the internet only through smartphones are significantly less likely to access patient portals than individuals with a wired connection [10].

Given the importance of patient portals for secure, expedient, and high-quality psychiatric care through video-based visits, it is important to evaluate patient portal access among behavioral health patients, as research on patient portal–based video visits in behavioral health settings has lagged behind similar research in primary care settings [18,21]. Early in the COVID-19 pandemic, our outpatient adult behavioral health clinic at an urban safety net hospital sought to assess patient preference for patient portal–based video visits or telephone-only visits since telepsychiatry had not been offered prior to the pandemic. Safety net hospital systems consist of hospitals and providers that deliver a substantial amount of their care to patients insured through Medicaid or who have no insurance. Historically, safety net hospitals have cared for vulnerable and underserved populations, often consisting of racial and ethnic minorities [22,23].

The aim of this study is to describe patient preference for telepsychiatry visits and evaluate for demographic characteristics associated with preference for video-based visits. Based on past research on patient portal use, we hypothesized that patients who have public insurance, are homeless, and are older will be less likely to choose video-based visits through the patient portal, due to diminished ability to access the required technology [10,14,20,24-27].

## Methods

### Study Design and Recruitment

Since telepsychiatry had not been offered prior to the pandemic, our outpatient adult behavioral health clinic at an urban safety net hospital conducted an 8-week outreach project to improve patient engagement with video visits through the hospital’s patient portal. This clinic is based at an academic medical center and serves a demographically diverse patient population. The clinic treats a range of psychiatric disorders, including mood disorders, anxiety disorders, trauma-based disorders, psychotic disorders, and substance use disorders. Outpatient services, including medication management, individual therapy, and groups, are available on site at the clinic. There is also a subspeciality clinic for psychosis that administers long-acting injectable antipsychotics and closely monitors patients on clozapine. Prior to the COVID-19 pandemic, visits only took place in-person, and in 2019, there were 7405 unique patients seen in the clinic between January and December. At the time of the outreach efforts, the majority of clinic visits were virtual, although face-to-face visits were still available, for example, for the antipsychotic injection clinic.

Between June 15 and August 21, 2020, clinic staff contacted all patients by telephone, both new and established, who were scheduled for an upcoming appointment in the clinic. Using a previously defined script, staff asked patients about their preference for future telepsychiatry visits—video based through the patient portal or telephone only. If a patient expressed preference for a video visit, they were asked whether they would use a smartphone or computer. For telephone-only visits, if a patient reported availability of a smart device or computer and internet access, this was also recorded. Data on preference for face-to-face visits was not collected, as the goal was to collect information on preference for the type of telepsychiatry visits.

For this study we included patients ≥18 years of age who demonstrated plans to continue care in the clinic with upcoming appointments. We used the information collected by clinic staff and conducted a retrospective chart review of the EMR to collect demographic information including age, sex, race, ethnicity, insurance, and homeless status. Ethnicity data was divided into two categories based on the EMR’s categorization, which divides ethnicity into “Yes-Hispanic or Latino” or “No-Not Hispanic or Latino.” Homeless status was determined through review of clinical documentation as well as a search of the EMR for the words “homeless,” “housing,” “lives,” and “stays.”

A total of 315 patients were called by clinic staff. Seven patients were excluded from the sample because they were not ≥18 years of age (n=1), were no longer receiving treatment in the clinic or informed clinic staff they were cancelling their upcoming appointments (n=4), or for whom a valid medical record number was not listed (n=2). Among the 308 patients included in the sample, clinic staff were able to contact 128 (41.6 %) patients. This study was approved by the Institutional Review Board.

### Statistical Analysis

We first stratified patients by those who clinic staff were and were not able to contact, and compared demographic characteristics between groups. We then limited our sample to those who clinic staff were able to contact and compared the rates of those who chose video-based visits through the patient portal between patients with different demographic characteristics. We used bivariate analyses in the form of *t* tests to analyze continuous outcomes and Pearson chi-square or Fisher exact tests to analyze categorical outcomes. Crude odds ratios were calculated based on the bivariate analyses. In the event of zero cells, we added 0.5 to all cells to calculate the odds ratio. Analyses were performed with Stata, Version 16.1 (StataCorp).

## Results

Patients who were contacted by clinic staff did not differ significantly from patients who the staff were not able to contact (n=180) with respect to age, sex, race, ethnicity, or homeless status (Table 1). However, there was a significant difference in insurance status between groups (*P*<.001). The group of patients who the clinic staff were able to contact had a greater percentage of patients with no insurance compared to the group that the clinic staff were unable to contact (n=11, 8% vs n=0, 0%).

Among the 128 patients that clinic staff were able to contact, 79 (61.7%) preferred video-based visits through the patient portal and 49 (38.3 %) preferred phone-only visits (Table 2). Among those who chose video-based visits, 55 (69.6%) patients preferred to access the patient portal through a smartphone, 16 (20.3%) through a computer, 3 (3.8%) through a tablet, and 5 (6.3%) did not have a preference documented. Among the 49 patients who preferred phone-only visits, clinic staff notes for 8 patients indicated that 2 did not have internet access, and 6 did not have access to a smart device or computer. The percentage of patients who chose video-based visits through the patient portal significantly differed by patient age group (Table 2). Patients who were ≥55 years of age were at 91% and 84% decreased odds of choosing video-based visits through MyChart when compared to patients 18-34 years of age and 35-54 years of age, respectively. There were no significant differences in preference for video visit through the patient portal by gender, race, ethnicity, insurance status, or housing status.

**Table 1:** Demographics characteristics of adult outpatient psychiatry clinic patients who clinic staff were and were not able to contact by telephone between June 15 and August 21, 2020.

		Not able to contact (n=180)	Able to contact (n=128)	*P* value
Age (years), mean (SD)		45.0 (13.5)	42.2 (14.2)	.08
**Age groups (years), n (%)**				.33
	18-34	54 (30)	44 (34)	
	35-54	78 (43)	59 (46)	
	≥55	48 (27)	25 (20)	
Male gender, n (%)		72 (40)	49 (38)	.76
**Race, n (%)**				.36
	White	75 (42)	42 (33)	
	Black/African American	64 (36)	48 (37)	
	Asian	2 (1)	1 (1)	
	Native American	0 (0)	2 (2)	
	Other (including Hispanic/Latino)	8 (4)	8 (6)	
	Declined	31 (17)	27 (21)	
**Ethnicity, n (%)**				.07
	Not Hispanic/Latino	148 (82)	94 (73)	
	Hispanic/Latino	31 (17)	30 (23)	
	Declined	1 (1)	4 (3)	
**Insurance, n (%)**				<.001
	Public (Medicare/Medicaid)	140 (78)	88 (69)	
	Private	40 (22)	29 (23)	
	Uninsured	0 (0)	11 (8)	
**Homeless, n (%)**				.67
	Not homeless	146 (81)	105 (82)	
	Homeless	23 (13)	18 (14)	
	Unknown	11 (6)	5 (4)	

**Table 2:** Demographic characteristics of adult outpatient psychiatry clinic patients who chose telepsychiatry visits by telephone or video through a patient portal.

		Telephone (n=49)	Video (n=79)	Video over telephone visits, odds ratio (95% CI)	*P* value
Age (years), mean (SD)		49.5 (2.1)	37.7 (11.7)	N/A^a^	<.001
**Age groups (years), n (row %)**					<.001
	18-34 (reference)	10 (23)	34 (77)	N/A	
	35-54	20 (34)	39 (66)	0.57 (0.24-1.39)	
	≥55	19 (76)	6 (24)	0.09 (0.03-0.30)	
**Gender, n (row %)**					.08
	Male (reference)	14 (29)	35 (71)	N/A	
	Female	35 (44)	44 (56)	1.47 (0.61-3.59)	
**Race, n (row %)**					.38
	White (reference)	18 (43)	24 (57)	N/A	
	Black/African American	19 (40)	29 (60)	1.44 (0.49-2.66)	
	Asian	0 (0)	1 (100)	N/A	
	Native American	2 (100)	0 (0)	N/A	
	Other (including Hispanic/Latino)	2 (25)	6 (75)	2.22 (0.34-24.96)	
	Declined	8 (30)	19 (70)	1.78 (0.64-4.98)	
**Ethnicity, n (row %)**					.94
	Not Hispanic/Latino (reference)	37 (39)	57 (61)	N/A	
	Hispanic/Latino	11 (37)	19 (63)	1.12 (0.48-2.62)	
	Declined	1 (25)	3 (75)	1.94 (0.15-104.95)	
**Insurance, n (row %)**					.67
	Public (Medicare/Medicaid; reference)	36 (41)	52 (59)	N/A	
	Private	9 (31)	20 (69)	1.54 (0.63-3.76)	
	Uninsured	4 (36)	7 (64)	1.21 (0.28-6.06)	
**Homeless, n (row %)**					.06
	Not homeless (reference)	39 (37)	66 (63)	N/A	
	Homeless	10 (56)	8 (44)	0.47 (0.17-1.30)	
	Unknown	0 (0)	5 (100)	6.53 (0.35-121.36)	

^a^N/A: not applicable.

## Discussion

### Principal Results

Results of an outreach project to adult outpatient behavioral health patients at a large urban safety net hospital during the COVID-19 pandemic and subsequent chart review showed that 61.7% (n=79) of patients contacted preferred telepsychiatry visits by video through the hospital’s patient portal to phone. We also found patients 55 years and older were 84% to 91% less likely to choose video-based visits through the hospital’s patient portal compared with younger patients ages 18-34 years and 35-54 years, respectively. Finally, among patients who chose video-based visits through the patient portal, 69.6% (n=55) preferred to use a smartphone.

### Comparison With Prior Work

Our main finding that 61.7% (n=79) of patients preferred video-based visits through the hospital’s patient portal to phone visits is lower than a similar survey during the COVID-19 pandemic of patients in a large hospital-based psychiatry clinic that found 82.8% of patients chose video-based visits through a patient portal [28]. The differences in preference for video-based visits over phone visits could be related to demographic differences between clinics that may reflect access to technology, clinic workflow, or other structural influences [29]. Notably, our sample included patients who primarily had public insurance (n=88, 69%) and had greater racial diversity (n=48, 37% Black, n=42, 33% White) compared to the prior study where patients primarily had private insurance (65.2%) and were largely White (77.5%) [28]. A recent survey of providers in a safety net hospital in a non–mental health setting showed results consistent with our study [29]. These providers reported that most visits were conducted over the telephone due to patient preference, with technological problems and digital literacy being the most common barriers to video visits [29]. Given the importance of video-based visits through patient portals for delivering safe [29], high-quality psychiatric care, increased research with demographically diverse patient samples is needed to identify what barriers may be leading to decreased preference for this type of telepsychiatry visit.

The finding that older patients in behavioral health were 84% to 91% less likely than younger patients to choose video visits through the hospital’s patient portal is consistent with the survey previously mentioned that was conducted during the COVID-19 pandemic with patients from a hospital-based psychiatry clinic [28]. Severe and colleagues [28] found patients 44 years and older were 1.2 times more likely than younger patients to choose telephone visits over video-based patient portal visits [28]. These findings are also similar to research done prior to the COVID-19 pandemic in the primary care setting that found older adults 65 years and older were less likely to use patient portal–based video visits than adults 18-44 years of age [30]. Previous research has identified that older adults are less likely to use patient portals due to issues with computer literacy, physical and cognitive limitations, and concerns regarding privacy [25,26,31]. Since video-based visits provide greater flexibility for patients with transportation barriers [5,7,30,32] and possible opportunities for increased caregiver or family involvement [2,33], additional research is needed to develop strategies to address barriers to older adult engagement in video-based patient portal visits.

Our finding that 69.6% (n=55) of people who chose video-based visits through a patient portal preferred to access them by smartphone is similar to another study conducted within another safety net health system, the Los Angeles County Department of Health Services, which found 70% of patients accessed the patient portal through a mobile device [24]. In contrast, other studies in health care systems with predominantly private payors have found patients most commonly access the patient portal through a desktop computer [24,27,34,35]. Differences in the type of device used to access patient portals may be due to income since patients with low incomes are more dependent on smartphones for online access, as broadband internet is an added expense [24,30,35]. Screen size is a key difference between mobile devices and desktop computers, as larger screen size is correlated with a greater sense of user control over the device and increased feelings of satisfaction when using the device, which may be due to greater ability to perceive affective stimuli on larger screens [36]. Therefore, it is important that the controls and designs of patient portal interfaces be optimized for all devices. This may help increase equitable access to video-based visits through patient portals for patients who use smartphones to access the platform.

### Limitations

Results from our study must be considered in light of its limitations. First, data regarding preference for other types of video visits was not collected as part of this initial outreach project, as the goal of this outreach was to improve engagement with video visits through the hospital’s patient portal. As a result, it was not possible to compare preference for video visits through a patient portal with video visits through another modality such as Doximity or Skype. Clinic staff were also unable to contact the majority of patients scheduled for appointments, which limits the generalizability of our findings across the clinic population. Nevertheless, demographic characteristics were extracted from the medical record, and the only difference found between patients who staff were and were not able to contact was in the number of patients without insurance. However, other clinical characteristics that may have impacted patient preference for video visits through the patient portal were not extracted from the medical record or collected by clinic staff, including prior duration of treatment, psychiatric diagnosis, employment status, income, and the availability of assistance from others. These characteristics will be important to examine in future research. This study also took place at a single site, which limits generalizability to clinics in other parts of the country. Furthermore, we were limited in our ability to determine why patients chose phone visits instead of video-based visits through the patient portal since the data collected did not consistently document whether patients were unable to use video-based visits since they could not access the technology or because they did not want to use this type of telepsychiatry visit. As a result, the exact number of individuals who are “smartphone dependent” could not be determined. Additionally, some of the variables we investigated, such as homeless status, had relatively few patients, which limited our statistical power to detect a difference. Lastly, due to the time-limited nature of the patient outreach, we were unable to examine any evolution of trends in patient portal-based video visits over the course of the pandemic.

### Conclusions

In summary, although the benefits of video-based visits through patient portals are well documented, there has been limited research on the use of this type of telepsychiatry visit in behavioral health, particularly in vulnerable populations in safety net health care systems. We found relatively low engagement in video-based visits through the hospital’s patient portal, particularly among older adults, when compared to a health care system serving patients with mostly private insurance. We also found that most patients preferred to access patient portals through their smartphone. Compared to previous studies, this paper adds to the existing literature around technological equity by exploring patient-level engagement with patient portals in a safety net population. There is currently limited research on patient preference for telepsychiatry modality in this specific patient demographic, particularly in a behavioral health setting. Clinicians, hospital administrators, and researchers should keep in mind that some patients may be particularly hesitant to obtain care through this modality, even with the previously discussed benefits. Thus, there is a need for additional research to evaluate ways to increase patient engagement with video-based visits through patient portals in a behavioral health setting, especially as these types of visits may remain prominent modalities even as in-person appointments resume [37].

## Abbreviations

EMR: electronic medical record
